# An industrial procedure for the intestinal permeability enhancement of acyclovir: in-vitro and histological evidence

**DOI:** 10.1038/s41598-023-47306-2

**Published:** 2023-11-16

**Authors:** Omar Y. Mady, Sara Mohsen Thabit, Suzan E. Abo Elnasr, Asmaa A. Hedaya

**Affiliations:** 1 Department of Pharmaceutical Technology, Faculty of Pharmacy, Tanta University, Tanta, Egypt; 2 Department of Histology, Faculty of Medicine, Tanta University, Tanta, Egypt

**Keywords:** Drug discovery, Drug delivery, Pharmaceutics, Target identification

## Abstract

Acyclovir, an antiviral drug, has low bioavailability due to its low permeability. Consequently, high drug doses and frequent administration are required. This study investigates the use of span 60, at different concentrations, as a granulating agent to enhance drug permeability using an industrial procedure on a pilot scale. The micromeritics, drug content, drug crystallinity, drug partition coefficient, and drug release of the produced formulations were examined. The findings revealed an enhanced drug partition coefficient, suggesting drug entrapment in the polar portion of span 60. The drug release profiles exhibited rapid and complete drug release. The improvement of the drug permeability was evaluated using a modified non-everted sac technique. Notably, drug permeability through the rabbit intestine significantly improved, as evidenced by various calculated permeation parameters, providing insights into the drug absorption mechanism. The widening of the paracellular pathway was observed through histological examination of the rabbit intestinal segment, which aligns with the drug absorption mechanism. The utilization of a paracellular pathway enhancer as a granulating agent holds promise as a strategy to enhance the oral bioavailability of class III drugs. Overall, this study presents a novel drug delivery approach to enhance drug permeation and bioavailability, with potential implications for other medications.

## Introduction

Acyclovir is a nucleoside antiviral drug used to treat herpes group of DNA viruses like herpes simplex virus type 1 (HSV-1), herpes simplex virus type 2 (HSV-2), and varicella-zoster virus (VZV)^1^. It can also prevent cytomegaly virus infections that occur after a transplant and severe complications of Epstein-barr virus^2^.

The mechanism of action of acyclovir as an antiviral drug is reported by binding to HSV-thymidine kinases and inactivating viral DNA polymerase, which inhibits viral replication^3^.

Existing treatment dosage forms using acyclovir include oral, parenteral, and topical forms, but topical therapy is less effective due to low skin permeability. Furthermore, oral drug delivery provides various advantages as it is the most preferred route, is non-invasive, patient-compliant, avoids pain, and is convenient for drug administration. However, its absorption from the gastrointestinal tract is slow, variable, and incomplete, with a low oral bioavailability of around 15% to 30%^4^. This limits its therapeutic potential, and using higher doses can cause toxicities and adverse reactions^5^.

The limits of the therapeutic potential of the oral administration of acyclovir are owing to its low permeability. It is classified according to the biopharmaceutical classification system (BCS) as a class-III drug (high solubility and low permeability)^6,7^. Also, the frequency of administration of acyclovir is high. The recommended dose of acyclovir is 200 mg or 400 mg, five times a day, depending on the type of infection^8^. Accordingly, any permeability enhancement will lead to the enhancement of the drug's bioavailability.

Different techniques like Nanomedicine^9^, liposomes^10^, niosomes^11^, solid lipid nanoparticles (SLNs)^12^, nanosuspension^13^, nanocrystals^14^, and dendrimers^15^ have been suggested to enhance the drug's permeability.

Certain pharmaceutical excipients (such as chitosan, dimethyl b-cyclodextrin (DM-b-CD), sodium caprate (Cap-Na), and sodium lauryl sulfate (SLS)) have been found to enhance the permeation of acyclovir by affecting the drug's paracellular pathway^16^.

Span 60 is a non-ionic surface-active agent. It is often used in combination with one or more of the tween products to achieve a desired hydrophilic-lipophilic balance (HLB) value^17^. Spans offer several advantages over ionic surfactants, including increased stability, formulating flexibility, and wider compatibility. They are stable in mild acids, alkalis, and electrolytes and do not react with ionic ingredients or active ingredients^18^.

Span 60 is also used in the food industry and has a recommended acceptable daily intake (ADI). The European Food Safety Authority (EFSA) changed the ADI of 25 mg/kg body weight by the Scientific Committee on food (SCF) in 1974 and established a group ADI of 10 mg/kg bw per-day for sorbitan esters (expressed as sorbitan) and ADI of sorbitan monostearate is 26 mg/kg bw per day^19^. Researchers have successfully prepared Span 60 microcapsules containing metformin using a rapid congealing technique and found a clear improvement of the drug permeability from the upper part of the rabbit small intestine^20^. The solid dispersion of metformin HCl in Span 60 showed a significant enhancement of the drug permeation in correlation with the drug’s pharmacodynamic effect^21^.

Granulation is a crucial process that involves enlarging particles through agglomeration in the production of pharmaceutical dosage forms, mostly in tablets and capsules. This technique converts small fine or coarse particles into large agglomerates called granules. To ensure a uniform distribution of each ingredient throughout the powder mixture, the process starts with an initial dry mixing of the necessary powder ingredients, including the active pharmaceutical ingredient (API)^22–24^.

In an industrial procedure, Mady et al.^25^ used span 60 to increase the permeability of metformin HCl, resulting in improving drug permeability and pharmacodynamic properties. There is a strong correlation between the drug's permeation profile and its pharmacodynamic effect. Therefore, the objective of this work is to enhance the permeability of acyclovir by granulating it with a paracellular pathway enhancer substance using a pharmaceutical industrial procedure. The study also considers the micromeritics properties of the products, the drug entrapment procedure, the partition coefficient, and the drug release profile. A modified procedure for the non-everted sac technique would be used for the drug permeation enhancement study. In addition, to elucidate the enhancement mechanism of the paracellular pathway enhancer, it was suggested to study the modification that occurred in the intestinal wall tissue structure of rabbits. In summary, the aim is to develop an industrial procedure for enhancing the intestinal permeability of acyclovir and demonstrate the achievement of this goal using in-vitro tests and histological evidence.

## Materials and methods

### Materials

Acyclovir is a gift from Sedico pharmaceutical company (Egypt), Sorbitan monostearate 60 of research grade (span 60) is purchased from Oxford, Lab Chem, Mumbai (India), n-octanol is purchased from Riedel–de haen ag seelze-hannover (Germany), disodium monohydrogen phosphate and potassium dihydrogen phosphate is purchased from El-Gomhoria Company for Chemicals, Tanta city branch (Egypt). All other reagents and chemicals used were of analytical reagent grade.

### Methods

#### Industrial granulation procedure of acyclovir

The industrial granulation procedure used is previously reported by the authors^25^. Exact weight of acyclovir and span 60 were weighed to have powders with a net weight of 20 g containing either 15%, 20%, or 25% of span 60. The weighed powders were physically mixed using a locally made planetary mixer shaft for 5 min with a stirring rate of 100 rpm at room temperature. The temperature was then increased to 80 °C while continuously stirring for 15 min and then gradually decreased while stirring until it reached room temperature. The products were grinding and sieving, and the sieved granules which passed from 500 µm were collected and stored for further investigation.

#### Characterization of the prepared acyclovir granules

##### Micromeritics properties

A constant weight of either pure drug or prepared granules carefully flowed onto the surface of a funnel and filled in a suitable measuring cylinder as a simulation to actually occur in a tablet filling machine. The powder volume was measured and considered as the bulk volume (V_0_). The measuring cylinder filled with the powder was tapped at constant condition till no decrease in its volume occurred and the powder volume was taken as tapped volume (V_t_). The different parameters of the granules were calculated according to Eqs. (1–4)^26,27^:

1 $${\text{Bulk density}} = \frac{{\text{weight of the sample in gram }}}{{{\text{bulk volume in cm}}^{{3}} { }\left( {{\text{v}}_{{\text{o}}} } \right)}}$$

2 $${\text{Tapped density}} = \frac{{\text{weight of the sample in gram }}}{{{\text{Tapped volume of the sample in cm }}^{{3}} }}$$

3 $${\text{Compressibility index}} = \frac{{{\text{tapped denisty}} - {\text{bulk denisty }}}}{{\text{tapped denisty }}} \times 100$$

4 $${\text{Hausner ratio}} = \frac{{\text{tapped denisty }}}{bulk denisty }$$

##### Instrumental methods of drug analysis

*Conduction of standard method of drug analysis and construction of a validated calibration curve* The weight of acyclovir (50 mg) dissolved in 0. 1N HCL. A solution of 10µg/ml was then prepared and used for a drug absorption scan in the UV region using a pure solvent as a blank. The λ max was determined. Different drug concentrations were prepared and used for construction of the drug calibration curve. The validation procedures were conducted according to the International Council of Harmonization (ICH)^28^. The standard required parameters for validation like linearity range, the limit of detection, the limit of quantitation, correlation coefficient, accuracy, and precision were studied.

*Determination of the actual drug content* The weight of granules of each prepared product theoretically containing 100 mg of acyclovir was taken and dissolved in 60 ml of 0.1N HCl using heat at 60 °C, then cooling, and the volume completed to 100 ml with the same solvent. Two ml of the prepared solution was taken and completed to 100 ml with the same solvent. The drug absorbance in the prepared solution was measured at 255 using 0.1N HCl as a blank. The procedure was carried out in triplicate. The mean of theoretical and actual drug content was calculated using the following Eqs. (5) and (6)^29^.

5 $${\text{Theoretical drug content }}\left( {{\text{TDC}}} \right) \; = \; \frac{{\text{Drug total }}}{{\left( {{\text{drug total}} + {\text{span }}60} \right)}} \times 100$$

6 $${\text{Actual drug content }}\left( {{\text{ADC}}} \right) = \frac{{\text{actual drug content total }}}{{\left( {{\text{drug total}} + {\text{span }}60} \right)}} \times 100$$

*Differential scanning calorimetry (DSC)* The solid-state of the drug in the prepared granules compared with the pure drug was assessed by carrying out a thermal analysis test (DSC) for the pure drug and different granule products. The apparatus used is TA Instruments- waters LLC (USA) supplied with Trios’ software. The heating cycle was 10 °C/min, ranging from 20 to 400 °C. The diagram provided by the equipment was used and different thermal parameters measured by the software were used for comparison and studying the solid-state entrapment of the drug.

##### Experimental determination of the acyclovir partition coefficients (Log P)

An amount of acyclovir (20 mg) and that containing the same amount of the drug from each granule was dissolved separately in 10 ml of n-octanol. Distilled water (20 mL) was added while stirring for 20 min. The prepared solution was then transferred into a separating funnel and stood for separation into two equilibrated phases for 24 h. The drug concentration in the aqueous phase was measured spectrophotometrically at 255 nm. The value of log P was calculated according to Eq. (7)^30^.

7 $${\text{Log p}} = \frac{{\text{Drug concentration in octanol }}}{{\text{Drug concentration in water}}} \times 100$$

##### Drug release profiles

The release profile of the pure drug and all drug-prepared granules were conducted according to the standard pharmacopeia method. The dissolution apparatus, Paddle USP dissolution apparatus, Type Dis 6000 (Copley Scientific, UK), was used with a stirring rate of 75 rpm and cell-maintained temperature at 37 ± 0.5 °C. An exact weight of 400 mg acyclovir or product granules that actually containing the same drug weight was added to 900 ml of phosphate buffer pH 6.8 dissolution media containing 1 ml of tween 80. At predetermined time intervals, 5 mL samples were withdrawn for analysis, and 5 mL of the fresh release medium was added to replenish each sample withdrawn. The withdrawn samples were filtered, and the drug absorbance was measured at 255 nm using UV/visible spectrophotometer (Thermo Fisher Scientific, model EVO 300PC, software: Vision Pro, USA). Three replicates were conducted.

##### Drug permeation profiles

The intestinal permeation profile of the pure drug and that from different granules were studied, Authors suggested a drug permeation enhancement study using a modified procedure from the non-everted sac technique^20^. To ensure the viability of the intestinal segment during the drug permeation profile study, Tyrode solution was used as an acceptor compartment since it was reported that the viability of the segment could be 36 h in Tyrode solution, which supplied with the requirements of carbon dioxide and oxygen^31^. Therefore, a validated calibration curve for acyclovir was conducted as reported in Section "Characterization of the prepared acyclovir granules". using Tyrode solution instead of 0.1N HCl.

*Preparation of the intestine sacs* The upper part of the intestine of a Male albino rabbit weighed 2 kg obtained from the Tanta animal house was used. All procedures were approved and regularly controlled by the Animal Ethics Committee of the Faculty of Pharmacy, Tanta University, Egypt (code: TP/RE/5/23M-0022), and all experiments were performed by the guidelines and regulations of this committee. All the procedures were also carried out in full accordance with the ARRIVE guidelines 2020. In addition, adequate care was taken to minimize pain and discomfort for animals. The small intestine of the sacrificed animal was taken after anesthetizing the animal with ketamine HCl, carefully washed, and cleaned with saline solution before being placed in the Tyrode solution. It should be also reported that the isolated intestine was used at once in the permeation profile study. Sacs nearly 14 cm long were taken and washed again with Tyrode solution to ensure the removal of any solid particles in the intestine. The segments were filled with Tyrode solution after being tied from both sides with a surgical thread to check the absence of any leaks from the filled segments and again being placed in Tyrode solution after emptying and filled with a perfusion drug solution.

*Preparation of perfusion solutions (Doner solutions) and filling the intestinal sacs* An exact weight of 20 mg of the drug or product granules containing the same amount of drug was dissolved in 10 ml of phosphate buffer pH 6.8 with or without increasing the temperature to 50 °C, cooling, and 1 ml of tween 80 was added while stirring. Then, 4 ml of the prepared solution was used to fill a previously prepared segment after emptying it from the Tyrode solution, then tied again with a surgical thread, and tested for any leaks. The filled segment length and diameter were measured for the calculation of the drug permeability coefficient.

*Studying the drug permeation profiles* The filled rabbit intestinal segment was suspended on the shaft of the USP dissolution apparatus. The media outside the sac (Permeation solution) was 900 ml of Tyrode solution. The suspended segment was rotated by rotating the shaft with a stirring rate of 75 rpm. The permeation media was maintained at 37 ± 0.5 °C. At predetermined time intervals, 5 ml was withdrawn and used to determine the drug concentration by measuring at 255 nm. The volume of the withdrawn solution was replenished by adding 5 ml of fresh media to maintain the volume of the permeation media at 900 ml. At the end of the permeation experiment of each test, the intestinal segments were carefully emptied from their content and fixed separately in a 2.5% phosphate-buffered glutaraldehyde solution obtained from the electron microscope unit (Faculty of Medicine, Tanta University, Egypt). The permeation profile study for each product was carried out in triplicates.

*Determination of the drug permeability coefficient* The drug permeability coefficient [apparent permeability] was determined according to the theoretical procedure reported^20,32,33^, The following equation, dM/dt = P S Cd, was used where dM/dt represents the moles of the solute transported per unit time, P is the permeability coefficient, S is the surface area of the membrane, and Cd is the concentration of solute in the donor. The value of M/SCd was calculated and plotted against time. The linear part of the curve was considered by linear regression analysis of the data and using a correlation coefficient value close to one. The slope of the linear part of the plot represents the permeability coefficient (P), with a velocity unit of (cm/s).

##### Histological visualization of the used intestinal segments

*Samples preparation for electron microscopic transmission* The tissues of the rabbit intestinal sacs used for drug permeation profile study were examined by using transmission electron microscopy (TEM) to investigate the ultrastructure of the tissue. At the end of the permeation profile study experiment, the sacs were carefully emptied from their contents and separatory immersed in 2.5% phosphate-buffered glutaraldehyde solution. Small specimens of the small intestine of each (1 mm^3^) were prepared for electron microscopic examination. They were fixed in 2.5% phosphate-buffered glutaraldehyde solution for 2 h at 4 °C (pH 7.4). The specimens were postfixed in 1% buffered osmium tetroxide solution for 1 h following phosphate-buffered saline washing. Then, the specimens were dehydrated through an ascending series of ethanol alcohol, treated with propylene oxide, and embedded in epoxy resin. After heat polymerization, the sections were cut using an ultramicrotome with a glass knife and were double stained with uranyl acetate and lead citrate^34^ to be examined using a JOEL (Columbia, SC, USA) electron microscope at the EM unit of the Faculty of Medicine, Tanta University.

*Morphometric study* Five different randomly selected fields from each ultrathin section of each sample were quantified using AMT Camera software linked to the transmission electron microscope and the intercellular spaces were measured.

*Statistical analysis* The estimated numbers were compared, and statistical analyses were performed using one-way analysis of variance (ANOVA) and Turkey’s test for group comparison. The mean and standard deviation were used to express all the data. If the probability P-value was less than 0.05, the differences were considered significant^35^.

### Ethics approval

All procedures of animal study were approved and regularly controlled by the Animal Ethics Committee of Faculty of Pharmacy, Tanta University, Egypt (code: TP/RE/5/23M-0022) and all experiments were performed in accordance with the guidelines and regulations of this committee. All the procedures were also carried out in full accordance with the ARRIVE guidelines 2020.

## Results and discussion

The purpose of this work is to introduce an industrial procedure for enhancing the permeability of the antiviral drug which is normally taken 5 times daily in 400 mg tablets (acyclovir)^36^. This hypothesis is based on the finding of the enhancement of metformin HCl permeability by applying the same procedure^25^. Both drugs (metformin HCl and acyclovir) are class III drugs according to BCS. The first applied technique was granulation with the paracellular permeability enhancement granulating agent (span 60). As an industrial procedure, it is essential to study the micromeritic properties of the prepared granules compared to the pure drug since the powder flow is a critical step in the manufacturing procedures. Different well-reported tests were conducted which were principally based on the bulk and tapped volumes of the tested powders and the results are summarized in Table 1. From the table, it can be noticed that the results of all applied methods are coincided with each other’s. The flow of pure drug is extremely poor which is slightly improved by granulation with span 60. Unfortunately, the flow property improvement of the drug by granulation is not enough for drug processing as a dosage form. Therefore, it is essential to add a gliding agent.

**Table 1:** Micromeritics properties of pure acyclovir drug and its granules with different span 60.

Substance	Compressibility index	Description	Hausner ratio	Description
Pure drug	42.37% (± 0.005)	Extremely poor	1.72 (± 0.014)	Very very poor
15% span 60	33.31% (± 0.019)	Poor	1.47 (± 0.057)	Very poor
20% span 60	31.50% (± 0.011)	Poor	1.46 (± 0.023)	Poor
25% span 60	22.53% (± 0.009)	Fair	1.29 (± 0.016)	Fair

A validated UV analysis method for the drug was conducted by dissolving the drug in 0.1N HCL, determining its λ max using 1 mg % (10 ug/ml), and the determined λ max was 255 nm^37^. A calibration curve of the drug was designed, with a correlation coefficient of 0.999 and linearity ranging from 0.04 to 1.6 mg%. Precision with RSD% values below 2%, and accuracy were confirmed by a recovery percent of 99.58 ± 1.9. The limit of detection was 0.0389, while the limit of quantification was 0.1181.

Table 2 shows the actual drug content which is very close to the theoretical drug content with minimal standard deviation. This is due to the granulation procedure used which also indicates the efficiency of the industrial procedure. There is no significant difference between TDC and ADC at *P* value > 0.05.

**Table 2:** The actual drug content in the prepared granules.

Span concentration (%)	TDC (%)	ADC (%)	SD ( ±)
15	85	85	0.859
20	80	80	0.852
25	75	74	0.967

**Figure 1:**
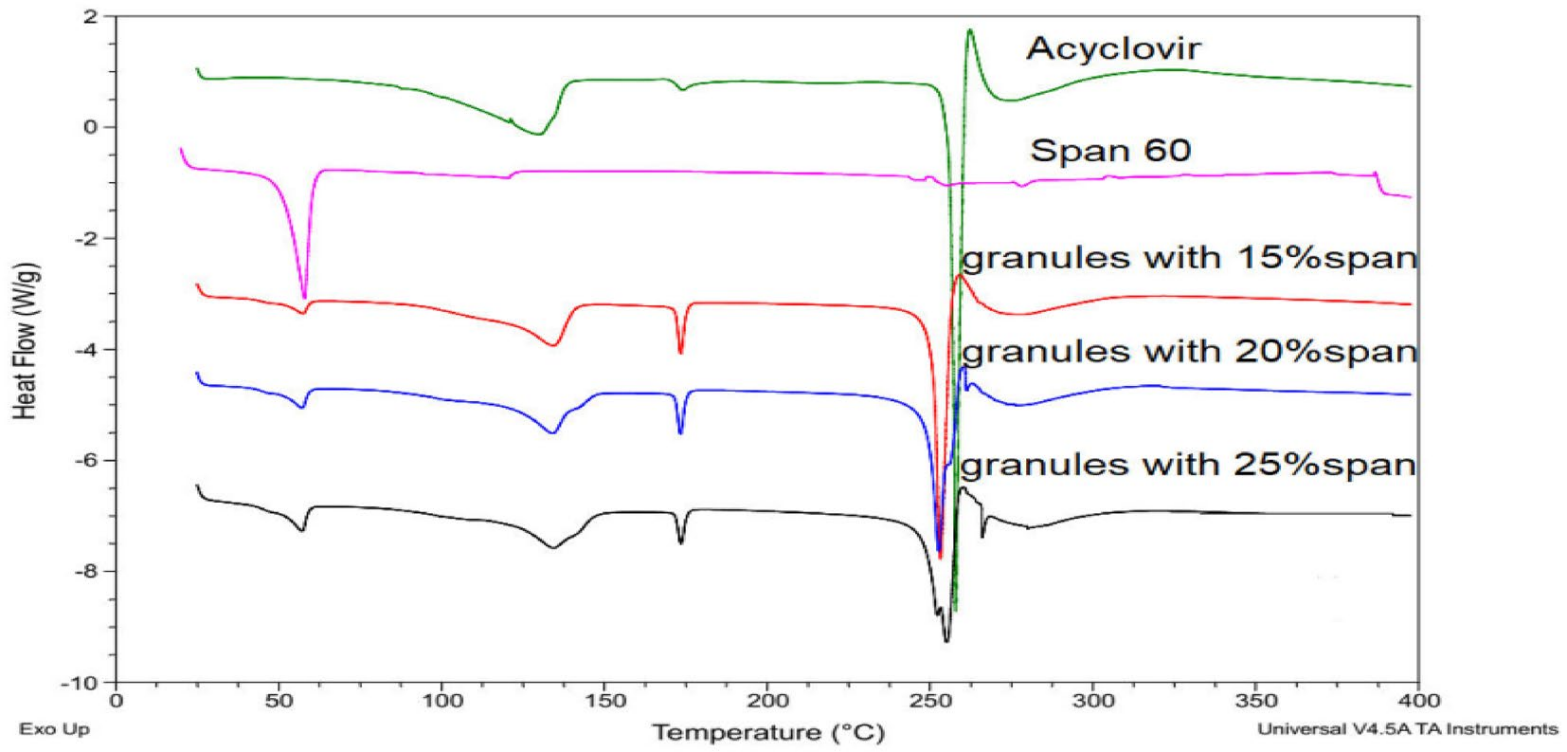
DSC scan of span 60, acyclovir and drug granules prepared by using different concentrations of the granulating agent.

The granulation procedure included increasing the temperature of the drug-span 60 physical mixture to 80 °C. Increasing the temperature of the physical mixture led to conducting a DSC scan to study the state of the drug in the granules. Figure 1 shows the results of the DSC scan for the drug, span 60, and the three-drug granules prepared with different concentrations of the granulating agent.

From Fig. 1. it can be noticed that the DSC scan of span 60 showed a sharp endothermic peak at 57.95 °C which represents the melting point of the granulating agent. The sharp endothermic peak of pure span 60 is changed to an endothermic broad peak with the same melting point and intensity, depending on the concentration of span 60 percent used.

At the same time, the DSC of the drug showed three peaks. The first was a broad endothermic peak with a melting point at 130 °C which represents the loss of the drug’s water of crystallization^38^. Young and Sang confirmed the loss of the drug's water of crystallization by storing the drug for 25 h at 0% RH (silica gel, 20) and carrying out a DSC scan of the anhydrous drug which showed a complete disappearance of the broad endothermic peak. The disappeared broad endothermic peak of the anhydrous drug rapidly appeared again on hydration of the anhydrous drug at 100% RH. In addition, the thermal gravity studies confirmed the loss of the drug's water of crystallization. The second is a minor endothermic peak between 170 and 175 °C with a melting point of 173 °C which represented a transition state of the drug, followed by a sharp melt-decomposition peak of the drug at 257 °C^39^.

The effect of the granulating agent on the DSC of the drug could be also studied. The shape of the first drug endothermic peak is changed with some kind of splitting, especially on using a 20% and 25% span. The drug transition peak at 170–175 °C looks sharper than that of the pure drug, and it is dependent on the concentration of span % used (increasing span concentration led to decreasing the peak intensity). The sharp drug endothermic peak has the same shape as the pure drug, but it shifts to a lower temperature on using 15% span as a granulating agent, which splits into two peaks on using 20% and 25% of span. The splitting of the drug sharp endothermic peak on using 20% span leads to a sharp one in the same position of the drug peak on using 15% span and a small second peak near the peak of the pure drug. When using 25% span, a sharp peak appears at the same position as the drug peak when using 15% span and a sharper one near the pure drug. These results may indicate the effect of molten span 60 as a surface-active agent on the drug's water of crystallization (peak 1), the drug transition state peak (peak 2) and the drug sharp endothermic peak (peak 3).

The DSC equipment is supplied with TRIOS software which is used for calculation of some thermal parameters listed in Table 3. From the table, it can be concluded that the presence of span 60 in the pure form in the granules prepared with different concentrations since there is no change in its melting point with irregular enthalpy values. That may be due to the lower melting point of span 60, the higher melting point of the drug, and the mechanism of the granulation process in general^25^. The melting point of the broad endothermic peak (peak 1) of the drug is shifted to a higher temperature with lower enthalpy values which decreases by increasing the concentration of span used. The minor endothermic peak of the drug (peak 2) can also be noticed at the same melting point of the pure drug (173 °C), but with a higher enthalpy value than that of the pure drug. The enthalpy value decreases by increasing span concentration. These software-calculated values for the drug's minor endothermic peak in the granules are completely consent with the intensity of the same peak reported in Fig. 3. The drug’s sharp endothermic peak (peak 3) is shifted to a lower temperature than that of the pure drug. The irregular melting point may be due to the splitting of the peak on using 20% and 25% span which is also reflected in the enthalpy values. In each case, the drug enthalpy value of peak 3 is markedly lower than that of the pure drug. From the DSC study, it can be reported the presence of the drug in its crystalline state^25^. The role of the granulating agent is only aggregation of the drug particles into granules by different reported mechanisms^25,40^. This conclusion is based on the presence of span 60 in its pure state and the high melting point of the drug. The modifications that were reported previously in the DSC of the drug in different products could be attributed to the molten span 60 which affects the drug's water of crystallization, transition state, and the drug crystalline endothermic peak. These conclusions are based on the high melting point of the drug and the nature of span 60 as an emulsifying agent.

**Table 3:** Thermal parameters of span 60 and acyclovir in pure form and granules.

	Peak 1		Peak 2		peak 3		Span 60	Peak
	mp °C	∆H(J/g)	mp °C	∆H(J/g)	mp °C	∆H(J/g)	mp °C	∆H(J/g)
Pure drug	130.3	145.8	173.9	5.2	257.8	187.4	57.95	86.38
15% span	134.4	86.82	173.4	12.9	253.1	143.6	57.42	10.12
20% span	134.1	62.09	173.4	10.99	252.7	144.2	57.13	16.52
25% span	134.7	54.36	173.5	8.92	255.1	141.5	57.17	11.86

**Figure 2:**
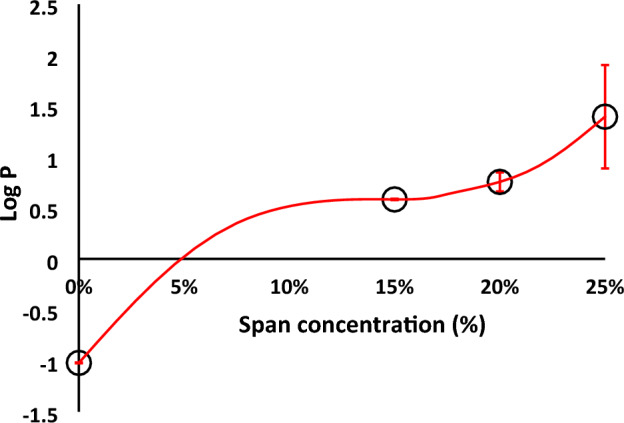
Partition coefficient of the drug and its granules between aqueous/n-octanol system.

Acyclovir is classified as a class III drug due to its high solubility and low permeability. The low drug permeability may be due to its hydrophilic nature. Decreasing the drug’s hydrophilicity and increasing its lipophilicity represents the basic philosophy for its granulation with span 60 using the melt method. To test the achievements of this aim, the partition coefficient of the pure drug and its products were carried out. The partition coefficient is a parameter used to measure the partitioning of the drug between two phases, the polar phase (normally water) and the non-polar phase (n-octanol). N-octanol is better for simulating the situation to a living tissue because it is like the membrane lipid. Figure 2 represents the Log P values against the concentration of the granulating agent used. From the Figure, it can be noticed the marked increase in the lipophilic property of the drug as a result of its granulation with span 60. The lipophilicity increased in a sigmoidal form indicating the huge increase by each addition of span 60. Span 60 was used as a granulating agent which was proven to cover the drug particles^20^. The addition of the drug product (granules) to the partition system (water / n-octanol) led to the entrapment of the drug in a special form of the non-ionic surfactant which diffused to the non-aqueous phase. This explanation is based on the experimented Log P values of the pure drug and the products which indicate the increase in lipophilicity of the drug as a result of its granulation by span 60.

### In-vitro drug release

**Figure 3:**
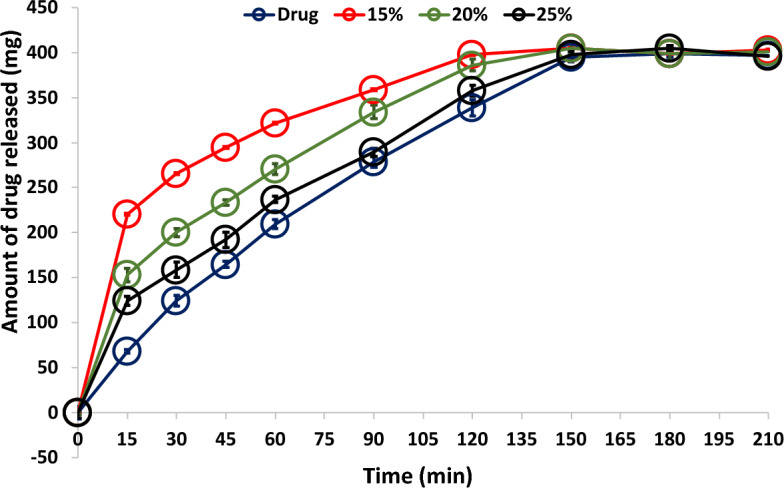
Acyclovir dissolution profile of pure drug and drug granules prepared with different span 60 concentrations.

Drug release profiles of acyclovir granules with varying concentrations of the granulating agent were studied in phosphate buffer pH 6.8 (Fig. 3). The results showed rapid initial drug release (burst effect) and different release rates. The release rate followed this order: 15% span > 20% span > 25% span > pure drug. Similar trends were observed for the burst effect. Complete drug release was achieved after 180 min in all cases. Mady et al.^25^ reported incomplete release and different release rates for metformin granules with a span 60. These release properties are dependent on span concentration due to its insolubility in the dissolution media. The addition of 1 ml of tween 80 to the dissolution media likely contributed to the complete release of acyclovir. The incomplete release of metformin^25^ and complete release of acyclovir with span granules suggest the role of tween 80 in the dissolution media. The lower dissolution profile of the pure drug, despite its smaller particle size, may be due to its lower solubility in the pH medium^41^. The complete solubility of 400 mg of pure acyclovir in the dissolution media, despite lower solubility in the pH medium, indicates the wettability effect of tween 80 on drug dissolution^41^.

The drug dissolution profiles showed a direct correlation with the concentration of span 60 used. This relationship impacts both the initial drug release from the granules and the drug release rates. These findings highlight the effectiveness of the suggested granulation process as a viable industrial procedure. Statistical analysis of the data (ANOVA test) revealed an insignificant difference in drug release at *P* > 0.05.

### Drug permeation profile

The intestinal sac is considered as a quick and sensitive technique for estimation of the intestinal permeability of the drugs. It is used for determining the overall intestinal integrity or comparative transport of a specific molecule, with the added benefit of the intestinal site of specificity. The apparent permeability [Papp] or permeation coefficient of a molecule through the intestinal barrier could be calculated^25,42^. The reported technique drawbacks are summarized by the author and discussed with suggestion for a solution to some of the technique's critical points. The suggested solution is to suspend the segment to the shaft of the dissolution apparatus. Therefore, the authors named it a modified non-everted sac which also led to the construction of a drug permeation profile instead of a drug release profile. In addition, the viability of the intestine segments was also considered by carrying out the experiment in Tyrode solution by supplying the medium containing the intestinal segments with their survival requirements of oxygen and carbon dioxide.

The volume of the perfusion solution filling the intestinal sac is low and contains a low amount of the drug. Therefore, the amount of the drug diffused to the acceptor could be expected to be low compared to the drug release profile study. In addition, the solution in which the experiment was carried out is completely different from that used for the drug release study which may affect the drug absorption. Accordingly, it was essential to carry out a valid calibration curve of the drug in Tyrode solution.

The drug scan showed the same drug λ max. the calibration curve had a correlation coefficient of 0.998, a linearity range of 0.02—0.9, a limit of detection of 0.0287, and a limit of quantification of 0.0872. The precision, as measured by RSD % values, was less than 2%, and accuracy was confirmed by a recovery percent of (102.312 ± 2.392).

**Figure 4:**
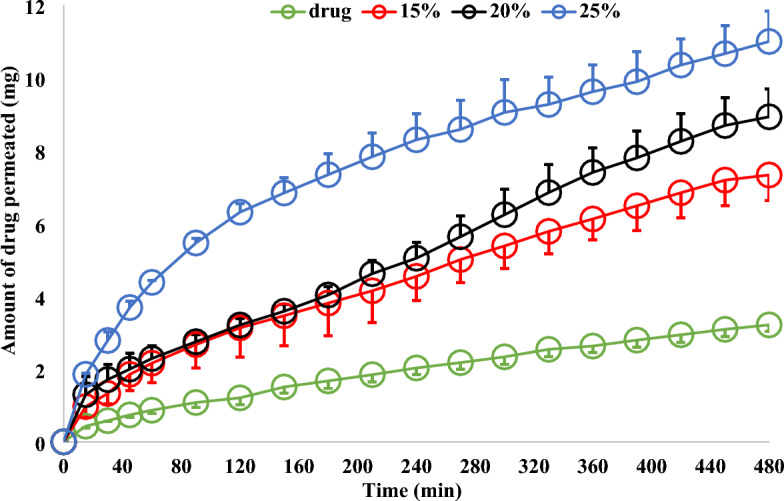
Acyclovir permeation profile from rabbit intestinal sacs by using modified non-everted sac technique.

Figure 4 shows the drug permeation profiles from the rabbit intestinal sac using the modified non-everted sac technique. From the Figure, it can be noticed that there is no rapid drug concentration permeated, in each case, which in the drug release profile is called the burst effect. The value of each represents the amount of drug permeated after 15 min. The initial rapid drug permeation process signalizes the drug transport mechanism through the paracellular route by passive diffusion^43,44^.

The total amount of drug permeated after 8 h, the drug permeation profile and the amount of rapid drug permeated could be arranged according to the following order: from drug granules prepared by using 25% Span ˃ from drug granules prepared by using 20% Span ˃ from drug granules prepared by using 15% span ˃ from pure drug. A huge drug permeation profile from the drug granules could be noticed by comparing it with that of the pure drug indicating the effect of the granulating agent on the drug permeation process. An overlap of drug permeation profile from granules prepared by using 15% and 20% granulating agent till 150 min could be noticed. A drug huge permeation profile difference from granules prepared by using 25% span from others is completely clear.

**Figure 5:**
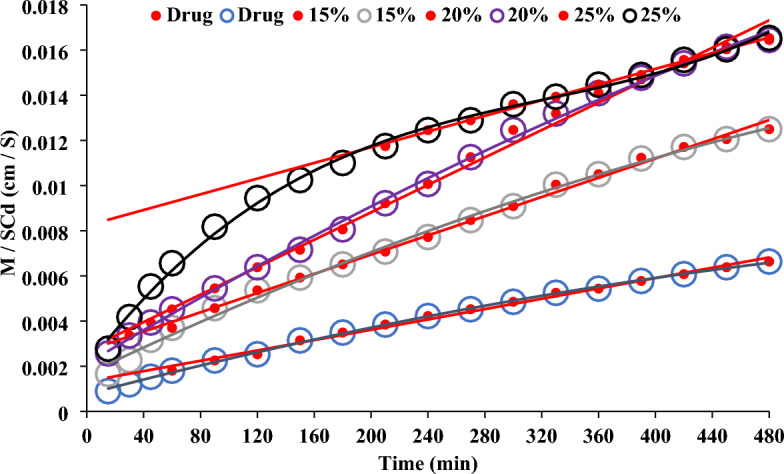
Determination the permeability coefficient of acyclovir from the upper part of the small intestine of rabbit.

Figure 5 represents the cumulative amount of drug transferred per unit concentration of the acyclovir in the mucosal solution as a function of time. It represents the relationship between M/SCd with a unit speed of (cm/s) and time (min)^33^. The curves in each case are fitted by a polynomial fitting model with a correlation coefficient of one. From the curve, it can be noticed that in each case there is no lag time indicating the absence of the transcellular drug absorption mechanism and supporting the reported paracellular pathway drug absorption mechanism^16,45^. The linear portion of the curve is determined by the value correlation coefficient. The values of the liner part at each time represent the independence of the drug permeability on the mucosal concentration of the drug, suggesting a passive transfer process. Then, the drug transfers constantly due to a constant concentration gradient across the intestinal barrier, which is maintained constant by replacing from the serosal fluid. The linear equation of the liner part is used for determining the drug permeability parameters. The drug permeability parameters are the lag time and the drug clearance^46^. The slope of the linear portion of the curve is used to calculate the drug permeability coefficient. The results are summarized in Table 4. From the table it can be noticed that the correlation coefficient values in each case are nearly one, indicating the steady state phase and a high correlation between the dependent and independent variables. From Fig. 5, in each case, the extrapolation of the linear segment to the abscissa intercepted with the y-axis in concentration which consents with the polynomial fitting of the drug permeation profile (Fig. 4) and cancels the transcellular drug absorption mechanism. Acyclovir is reported to be absorbed via the paracellular pathway. Therefore, the values of the intercept of the extrapolation in Table 4. may represent the concentration of the drug required to saturate the paracellular pathway at zero time^21,25,45^. There are irregular intercept values. Mady et al.^25^ found the value of intercept on using 20% span is higher than that on using 5% span as a granulating agent. However, this was only observed in the solid dispersion of the drug in span 60. The solid dispersion^21^ or molecular dispersion^20^ of the drug in span 60 led to a nearly constant intercept value. The granulation technique is an irregular dispersion of the granulating agent around the drug particles to form large granules for special manufacturing purposes, which has been previously proved by a scanning electron microscope^25^. At the same time, solid dispersion of the drug in the matrix may lead to a homogenous distribution of the drug in the matrix. Based on these two facts, the irregular intercept results may indicate the role of span on the structure of the paracellular pathway, especially if the reported finding about the paracellular pathway absorption of metformin is a saturable mechanism considered^45^. In addition, the use of 25% span as a granulating agent led to a huge increase in its intercept value (Table 4). The huge value of the intercept on using 25% span 60 is accompanied by lowering the drug permeation coefficient after regular permeation coefficient values increased with increasing the span 60 concentration (Table 4 and Fig. 5).

**Table 4:** Data of acyclovir transferred through modified non-everted intestinal sac (n = 3).

	r2	Intercept × 10^−3^	Papp (cm/s) × 10^−6^	Total penetration %	DAE%
Pure drug	0.999	1.3	10 (± 1.84)	16.055 (± 0.92)	100.000
15%span 60	0.996	2.7	20 (± 1.35)	35.630 (± 1.08)	221.924
20%span 60	0.994	2.7	30 (± 1.66)	41.125 (± 2.65)	256.151
25%span 60	0.997	8.2	20 (± 2.79)	54.960 (± 3.93)	342.323

DAE % = drug absorption enhancement percent compared to the pure drug.

Although there is an irregularity in the drug concentration required to saturate the paracellular pathway and permeation coefficient values, the total drug permeation percent and drug absorption enhancement percent (DAE%) increased by increasing the concentration of the granulating agent used (Table 4). The drug absorption enhancement percent (DAE%) is a suggested formula by the authors^20,21,25^. It represents the percent of drug absorption enhanced as a result of its granulation with span 60 considering the amount of the pure drug absorbed is 100%. It is calculated as the following Eq. (8):

8 $${\text{\% DAE}} = \frac{{\text{The cumulative amount of drug penetrated from the dosage form}}}{{\text{The cumulative amount of pure drug penetrated}}} { } \times { }100$$

The effect of increasing the granulating agent on the two parameters (total penetration percent and total drug absorption enhancement percent) could be arranged as the following: from granules prepared on using 25% ˃ on using 20% ˃ on using 15% granulating agent. These results conform with the previously reported by the authors on applying the same procedure on metformin HCl^25^.

**Figure 6:**
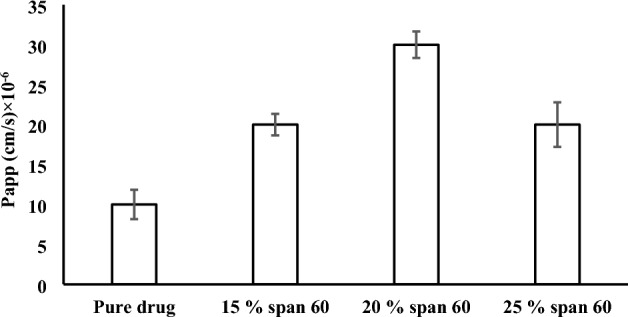
The apparent permeability of acyclovir (mucosal -to- serosal) from pure drug and the granulation products (n = 3).

Figure 6 represents the intestinal permeability coefficient enhancement effect of the granulating agent to the drug. From the figure, it can be noticed the increase in the drug’s apparent permeability as a result of its granulation with span 60 to 20% used and then decreased on using 25%.

The drug absorption enhancement effect was experimentally and mathematically proved through the effect of the granulating agent on the drug intestinal paracellular pathway. To visualize the effect of the granulating agent on the drug paracellular pathway, at the end of the drug permeation profile experiment, the intestinal segment was subjected to histological study to visualize the viability of the segment, histological modification, and the paracellular pathway extension.

**Figure 7:**
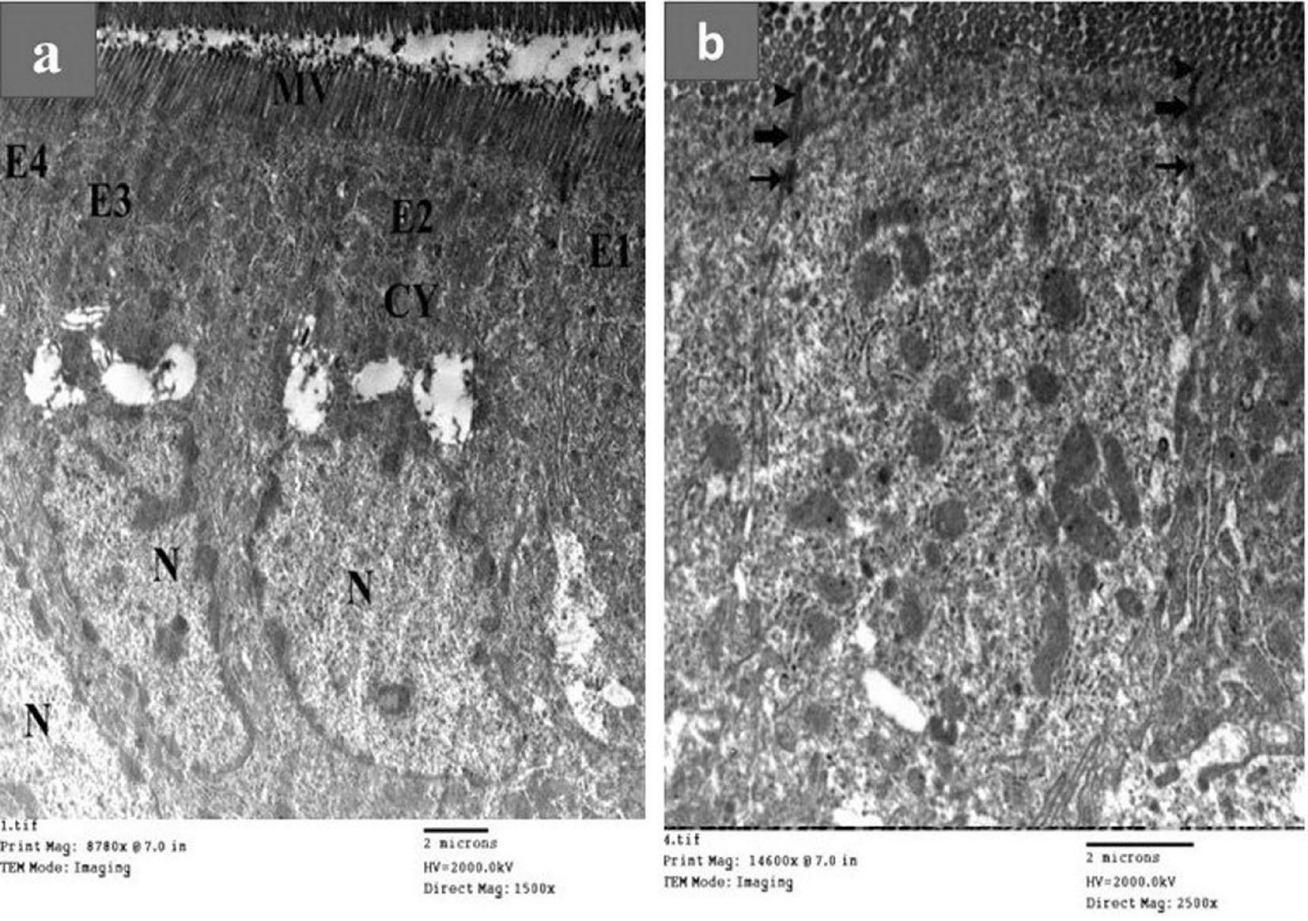
Electron micrographs of ultrathin sections in the small intestine of pure drug (Acyclovir). (**a**) (Mic. Mag × 1500). (**b**) (Mic. Mag × 2500).

Figure 7 represents the electron micrographs of ultrathin sections in the small intestine of pure drug (Acyclovir) showing the main cells forming the lining of the small intestine called absorptive columnar cells (enterocytes). (a) In this ultrathin section, there are four cells (E1, E2, E3 & E4). The cells have apical closely packed finger projections called microvilli (MV). Each enterocyte has a cytoplasm (CY) having vital organelles as mitochondria and nucleus (N). (b) Between each two adjacent cells, there are junctional complexes. Just below the base of the microvilli (MV), the cell membranes of adjacent cells are in close contact, and the intercellular space becomes zero at the tight junction (arrowheads), followed by the zonula adherens (thick arrows) at which the intercellular space becomes normal and then desmosomes (thin arrows) characterized by wider intercellular space.

**Figure 8:**
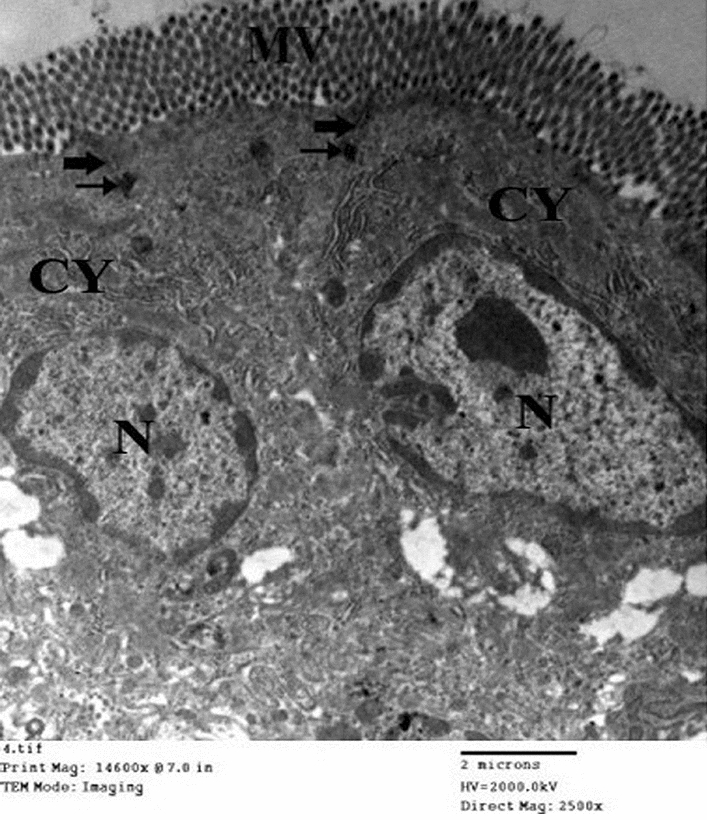
Electron micrograph of an ultrathin section in the small intestine of drug granules prepared on using 15% span 60 (Mic. Mag × 2500).

Figure 8 represents the electron micrograph of an ultrathin section in the small intestine of drug granules prepared by using 15% span 60 showing the enterocytes with apical microvilli (MV). Enterocytes appear with nearly normal cytoplasm (CY) and nuclei (N). Slight widening of the intercellular spaces is observed (thick and thin arrows).

**Figure 9:**
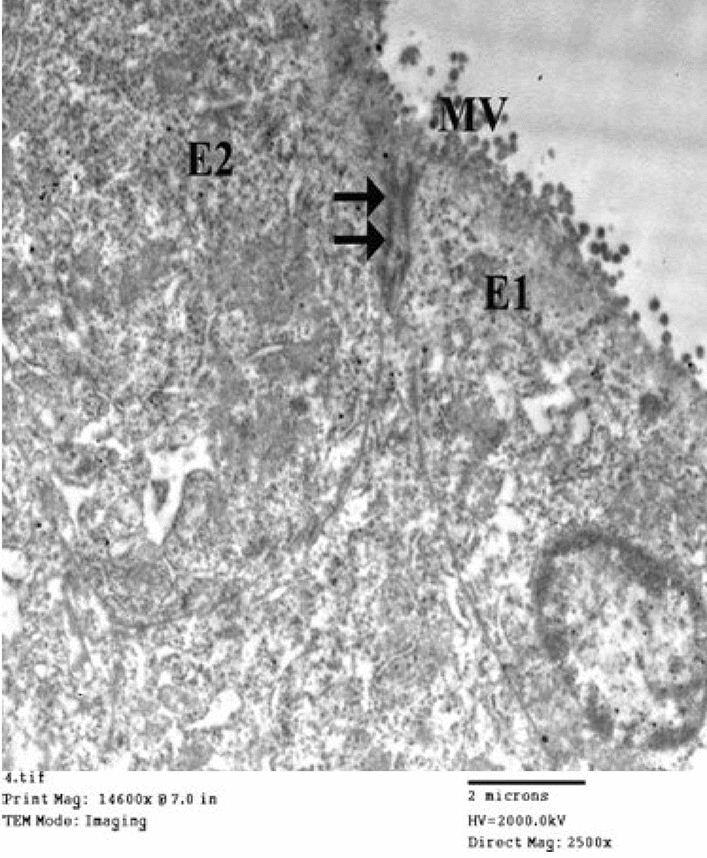
Electron micrograph of an ultrathin section in the small intestine of drug granules prepared on using 20% span 60 (Mic. Mag × 2500).

Figure 9 shows the electron micrograph of an ultrathin section in the small intestine of drug granules prepared by using 20% span 60 showing two enterocytes (E1 and E2) with apical microvilli (MV). No apparent cytotoxic changes are observed. The apparent widening of the intercellular spaces is also noticed (arrows).

**Figure 10:**
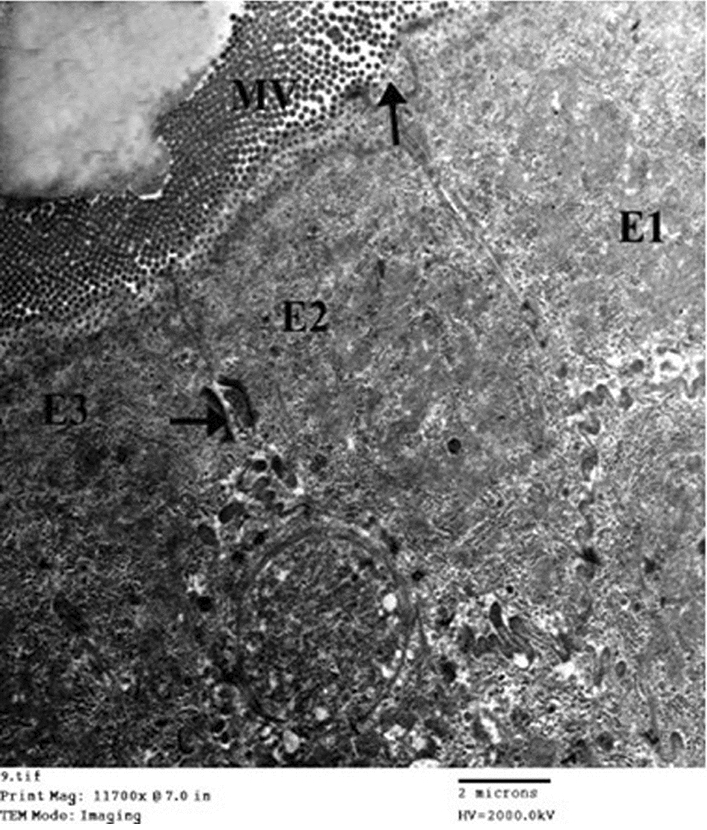
Electron micrograph of an ultrathin section in the small intestine of drug granules prepared on using 25% span 60 (Mic. Mag × 2000).

Figure 10 represents the electron micrograph of an ultrathin section in the small intestine of drug granules prepared by using 25% span 60 showing three enterocytes (E1, E2, and E3) with apical microvilli (MV). No apparent cytotoxic changes are observed. Apparently, more widening of the intercellular spaces is also observed (arrows).

### Statistical results

**Figure 11:**
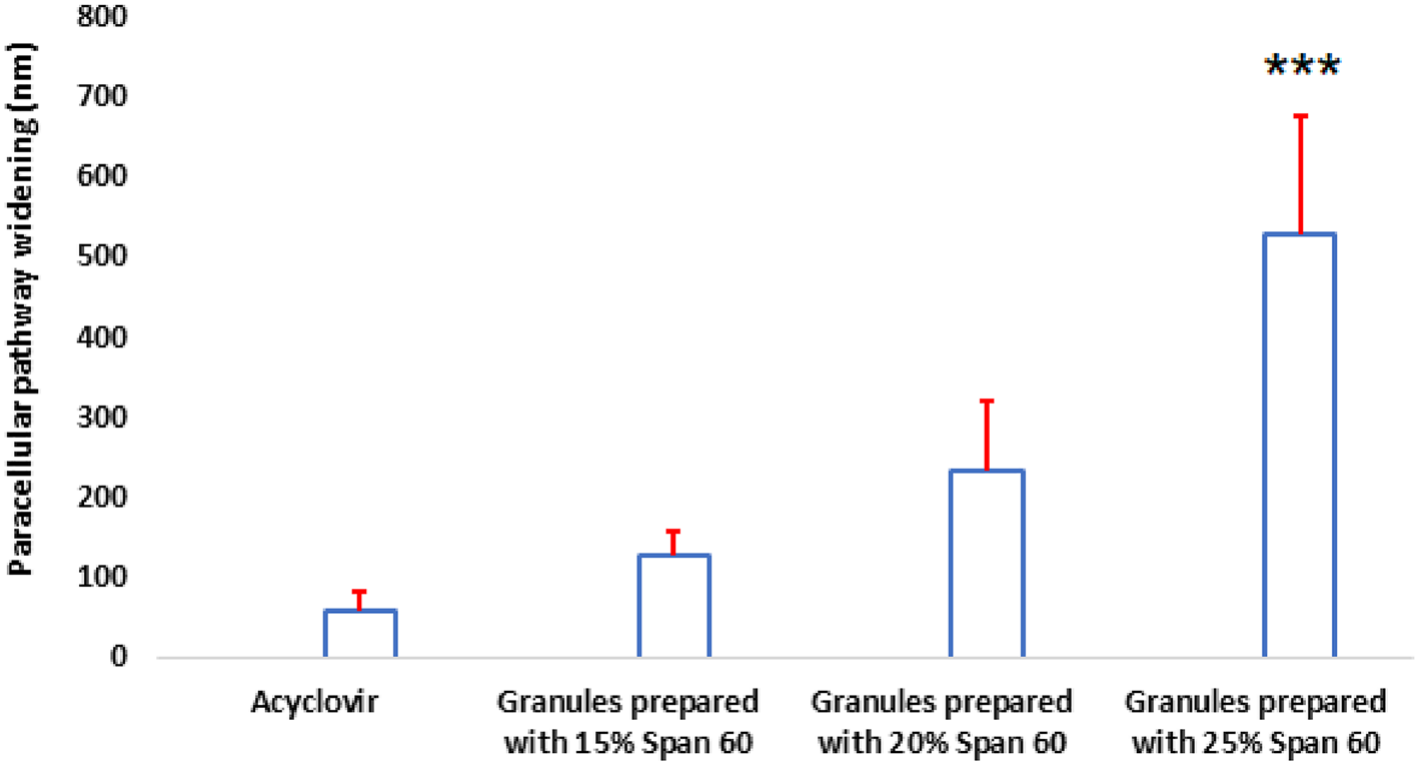
Histogram of the mean measured widening length (nm) of the paracellular pathway of the rabbit intestine segments on using different items. *** Highly significant.

The estimated widening numbers of the paracellular pathway as a result of the pure drug and drug granulated with different concentrations of span 60 were compared and subjected to a statistical analysis. The results showed a highly significant increase in the mean widening length of the intercellular space on using 25% span 60 as a granulating agent, compared to using either pure drug, 15% span 60, or 20% span 60 as a granulating agent at (*p* < 0.001) (Table 5 and Fig. 11).

**Table 5:** Mean measured widening length (nm) of the paracellular pathway of the rabbit intestine segments on using different items.

Items	Mean intracellular space (nm)	SD
Acyclovir	60.61	± 22.7
Granules prepared with 15% Span 60	127.42	± 31.6
Granules prepared with 20% Span 60	233.05	± 88.6
Granules prepared with 25% Span 60	528.17	± 148.4

The drug permeation profile study proved the intestinal permeation enhancement effect of span 60 for acyclovir. This could be noticed from the total drug permeation percent (Table 4). To clarify the role of the granulating agent on the drug permeation enhancement, the author suggested another item which is drug absorption enhancement percent (DAE%)^21^. Table 4 highlights the discernible impact of increasing the concentration of Span 60 as a granulation agent on both the total drug permeation and the DAE%. These improvements are directly linked to the varying levels of Span 60 concentration employed. It is reported that acyclovir is a class III drug absorbed mainly via a paracellular pathway with a diffusion saturation mechanism^16^. At the same time, it was proved that span 60 enhanced the permeability of metformin (a class III drug) via a paracellular pathway^21,25^.

A histological study of the intestinal sac at the end of carrying out the drug permeation profile visualized the widening of the paracellular pathway in the sac segment used for pure drug. This result consents with the reported paracellular pathway drug absorption mechanism. The widening of the paracellular pathway was significantly increased when the drug was granulated with span 60, suggesting that span 60 enhanced the permeation mechanism of the drug via widening its absorption pathway and it is also span 60 concentration dependent. Therefore, it can be concluded that increasing the span 60 concentration led to increasing the widening of the paracellular pathway and consequently increased the total drug permeation percent and DAE%. This visualized fact of the drug absorption mechanism which is enhanced by using span 60 supports the theoretical suggestion by the author about the use of a permeation profile curve for the prediction of the drug mechanism and the extension of the straight line to the intercept with y-axis in concentration representing the amount of the drug required to saturate the paracellular pathway.

Regarding the huge intercept value of the extended line to the y axis on using 25% span 60 as a granulating agent, the histological study of the sac segment showed a highly significant increase of the widening of the paracellular pathway. This again gives confidence in using the permeability profile curve.

The linear part of the drug permeability profile is used for the estimation of the drug permeability parameters. The slope of that line is the drug permeation coefficient. The apparent permeability of the drug on using 25% span as a granulating agent is decreased again to be equal to that on using 15% Span 60 although the total drug penetration percent and DAE% maintained the increasing order effect (Table 4). Figure 5 shows the deviation of the steady state part of the drug permeation process (straight line part) from the total drug permeation profile (polynomial part). Based on the correlation coefficient values, the linear steady state part was selected. Then, it can suggest the start point (time) of the steady state of the curve. For the drug, the steady state started after 45 min, while that for granules prepared on using 15% span 60 started after 45 min, and that for granules prepared by using 20% span 60 started after 15 min. At the same time, the use of 25% span 60 led to the extent effect of the paracellular pathway which delayed the start point of the drug’s steady state and may also change the drug permeability coefficient. The above theoretical reports, which are based on the drug permeability steady state are correlated with the widening of the paracellular pathway, visualized by studying the intestinal segment at the end of the drug permeation profile experiment. These results may prove the trust of using the suggested modified non-everted sac for studying the drug permeability, which may also give an idea about the mechanism of the drug absorption mechanism.

## Conclusion

The above work represents the dual benefits of an industrial procedure for a BCS class III drug. Dual benefits are concerned with the granulation process that also increasing the drug permeability, and consequently bioavailability. It is the second trial for BCS class III drugs to visualize the widening effect of the granulating agent on the paracellular pathway for drug permeation process. Therefore, it can suggest the procedure for other BCS class III drugs and also suggest the same procedure for other BCS classes which face permeability problems. This suggestion is based on two facts: first, the granulating agent used is span 60, which is used normally in the food industry with a higher consuming concentration than that in tablets prepared with the granulation procedure process. Second, the thermoplastic granulation technique is one of the widely used techniques for the drug tableting process due to its low cost and time effectiveness. Additionally, this technique can be applied to a large number of thermostable drugs up to the melting point of the granulating agent.

## Data Availability

The datasets generated during the current study are not publicly available because the second part of the research has not yet been finished or published. However, they are available from the corresponding author on reasonable request.
